# The Deep Atmospheric Boundary Layer and Its Significance to the Stratosphere and Troposphere Exchange over the Tibetan Plateau

**DOI:** 10.1371/journal.pone.0056909

**Published:** 2013-02-25

**Authors:** Xuelong Chen, Juan A. Añel, Zhongbo Su, Laura de la Torre, Hennie Kelder, Jacob van Peet, Yaoming Ma

**Affiliations:** 1 Key Laboratory of Tibetan Environment Changes and Land Surface Processes, Institute of Tibetan Plateau Research, Chinese Academy of Sciences, Beijing, China; 2 Faculty of Geo-Information Science and Earth Observation, University of Twente, Enschede, The Netherlands; 3 Smith School of Enterprise and the Environment, University of Oxford, Oxford, United Kingdom; 4 EPhysLab, Fac. of Sciences, Universidade de Vigo, Ourense, Spain; 5 Eindhoven University, Eindhoven, The Netherlands; 6 Royal Netherlands Meteorological Institute (KNMI), Utricht, The Netherlands; Plymouth University, United Kingdom

## Abstract

In this study the depth of the atmospheric boundary layer (ABL) over the Tibetan Plateau was measured during a regional radiosonde observation campaign in 2008 and found to be deeper than indicated by previously measurements. Results indicate that during fair weather conditions on winter days, the top of the mixed layers can be up to 5 km above the ground (9.4 km above sea level). Measurements also show that the depth of the ABL is quite distinct for three different periods (winter, monsoon-onset, and monsoon seasons). Turbulence at the top of a deep mixing layer can rise up to the upper troposphere. As a consequence, as confirmed by trajectory analysis, interaction occurs between deep ABLs and the low tropopause during winter over the Tibetan Plateau.

## Introduction

The Tibetan Plateau, or the “Roof of the World” [1], [2], is the world’s largest and highest plateau. It exerts a profound thermal and dynamical influence on the local atmosphere [3], and atmospheric processes above the plateau are crucial for regional studies of climate and weather. Research indicates that the evolution of the atmospheric boundary layer (ABL) over elevated terrain can influence its development in its near and downstream regions [4]. In particular, the plateau’s ABL influences its Eastern downstream area [5], [6], and some synoptic systems over East China originate in the ABL over the plateau [7], [8]. Convections over the Tibetan Plateau can influence the hydration of the global stratosphere [9]. A clear understanding of the ABL in this region is key to attempts to obtain a better picture of the thermodynamic influence of the plateau on weather and climate in Asia [10], [11].

Over the past thirty years, several comprehensive observational experiments have revealed important characteristics of the plateau’s land surface processes and of the structure of the ABL over the plateau [3], [12]–[17]. It is now known, for example, that the ABL over the plateau is deeper than that over the lowlands [3], [15], [18], [19]. However, few studies have investigated the connection of the high ABL with the upper troposphere and lower stratosphere (UTLS), even though the Tibetan Plateau is regarded to be a pathway of mass exchange between the troposphere and stratosphere [20].

The elevation of the Tibetan Plateau varies between 3000 and 8848 m above sea level (ASL). The top of the ABL may be as high as 9000 m ASL, which is close to the location of the tropopause: this is a result of both the plateau’s elevation and the depth of the ABL. This can result in a stronger interaction between the UTLS and the ABL than in lowland areas. Chen et al. [21] concluded that the Tibetan Plateau is one of three key source regions for transport from the boundary layer to the tropopause in the Asian monsoon region. Studies have shown that multi-tropopause events, which are closely related to tropopause folds, frequently occur over the Tibetan Plateau [22]–[24]. Tropopause folds can cause stratospheric air to be transported downwards to the ABL through a number of different processes [25]. A previous study indicated that dynamic transport was the main factor that influences the vertical distribution of ozone over the Tibetan Plateau [26]. Surface ozone over the Tibetan Plateau is sensitive to ozone perturbation in the upper layers [27], suggesting that ozone above the planetary boundary layer may strongly influence surface ozone. It is clear that the interaction between the plateau’s ABL and the UTLS is also important for troposphere-stratosphere exchanges.

The depth of the ABL changes in both space and time, and varies from less than one hundred to several thousand meters [28], [29]. To present our investigation of this behavior, this paper is organized as follows. The next section presents the data and methods used to derive the depth of the ABL. Next, the depth is analyzed as a function of season and tropopause height, based on intensive observations during the winter, monsoon-onset and monsoon seasons. The next section describes simultaneous variations in both tropopause folds and the ABL, and is followed by a Lagrangian analysis to determine the possibility of air mass exchange between a deep ABL and the UTLS. Finally, the discussion and conclusions are presented.

## Data and Methods

In a joint Sino–Japanese project, radiosonde observations were carried out over the Tibetan Plateau in 2008 [2]. The sounding dataset of the Gerze station (32.17°N, 84.03°E, 4415 m above mean sea level) was chosen to compare the ABL depth and tropopause height over the plateau, because of the absence of high mountains in the vicinity. In 2008, three intensive observation periods (IOPs) were used. The detail information about each IOP is listed in table 1. Vaisala RS-92 radiosondes were released every day at 01∶00, 07∶00, 13∶00 and 19∶00 BST (Beijing Standard Time, UTC+8) during these IOPs.

**Table 1:** Observation period for each IOP and its representative season.

	IOP1	IOP2	IOP3
Observation date	25, Feb-19, Mar	13, May-12, Jun	07, Jul-16, Jul
Representative season	Winter	Monsoon-onset	Monsoon

The height of the tropopause was determined using the World Meteorological Organization (WMO) lapse rate tropopause (LRT) definition [30], which defines the tropopause to be the lowest level at which the lapse rate decreases to 2 K/km or less, provided that the average lapse rate between this level and all higher levels within 2 km does not exceed 2 K/km.

The top of the convective boundary layer (CBL) was determined using the simple parcel method, which is a reliable method in unstable conditions [31]–[33]. This method equates the top of the CBL with the intersection of the actual potential temperature profile with the dry-adiabatic ascent, starting at ‘near-surface temperature’.

The ECMWF ERA-Interim data [34] was used to analyze the UTLS structures around the plateau during the radiosonde ascents. Trajectory models are often used to trace the movement of air particles (e.g. Ladstätter-Weißenmayer et al. [35], Bergman et al. [36]). In order to check possible exchanges between the high CBL and the stratosphere, a trajectory model forced with ERA-Interim data was used to simulate whether the air mass can be transported from troposphere to stratosphere, and vice versa. The FLEXPART v8.1 model [37], [38] was used to perform the trajectory analysis.

Ozone profiles from the GOME-2 instrument [39] were also used to study the structure of the UTLS from space. GOME-2 measures backscattered solar radiation from the Earth’s atmosphere between 250 and 790 nm in four channels, with a relatively high spectral resolution (0.2–0.4 nm). In its normal mode, the instrument has an almost global daily coverage with a cross-track swath width of 1920 km, split into ground pixels of size 640×40 km. Ozone profiles were retrieved using the optimal estimation method described in Rodgers [40], with a pressure grid of 41 levels between the surface and the top of the atmosphere.

## Results

### The Variations of ABL Depth and Tropopause Height

**Figure 1:**
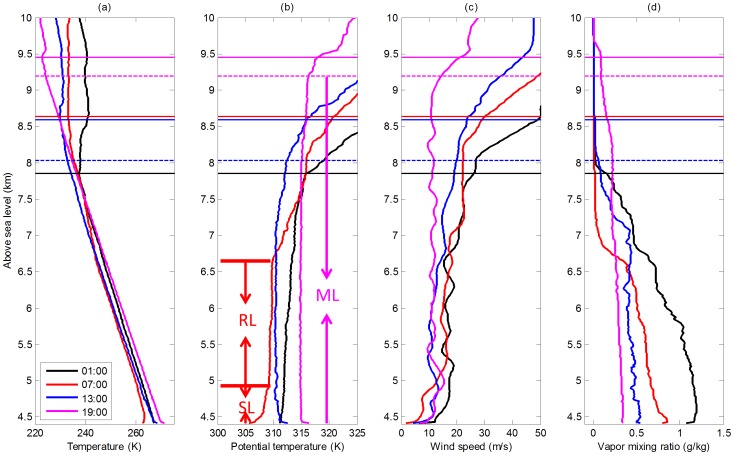
Profiles of (a) temperature, (b) potential temperature, (c) wind speed, (d) water vapor content on 25 Feb 2008. Profiles were recorded at 01∶00 (dark line), 07∶00 (red line), 13∶00 (blue line), and 19∶00 (magenta line) BST. The horizontal dashed lines show the corresponding tops of the CBL, and horizontal solid lines show the positions of the tropopause. The stable layer (SL), residual layer (RL), and mixed layer (ML) are also marked.

According to meteorological measurements, IOP1, IOP2 and IOP3 represent winter time, monsoon-onset, and monsoon time respectively. From IOP1, 25 February was chosen as a typical sunny day, in order to analyze the detailed structure of a very deep ABL (Fig. 1). A surface-based inversion formed during the early morning, which was eliminated by 13∶00 (Fig. 1a). The 07∶00 sounding revealed a cool, stable layer (SL) 500 m deep near the surface. Above this was a residual layer (RL) (Fig. 1b) of 1.7 km depth, which had a nearly constant potential temperature. After sunrise, the net radiation quickly increased, causing the surface to warm rapidly. By 13∶00, this surface heating had caused sufficient growth of the mixed layer (ML) to replace the stable surface layer and residual layer. By 19∶00, a mixed layer 4780 m high had been formed (equal to 9195 m ASL). Despite the fast development of the mixed layer, the water vapor and wind speed were also well mixed by turbulence, as generated by surface heating (Fig. 1c and 1d). ABL growth also entrained relatively dry air from above, which reduced the water content of the mixed layer between 01∶00 and 19∶00. The water vapor content in the mixed layer decreased slightly with increasing height, due to surface evaporation. All these phenomena suggest a mixed layer dominated by convection.

The wind shear in the troposphere on 25 February was weaker than on other days. At around 9.3 km ASL the wind speed increased very quickly, and the water vapor content dropped quickly. The layers of high gradient around 9.3 km ASL can be regarded as the top of the CBL, because of the well mixed layers below this height, or as an indication of the UTLS. Using the definitions mentioned previously, the top of the CBL and the tropopause height are indicated by horizontal dashed lines and solid lines respectively. From 01∶00 to 19∶00, the height of the tropopause and of the top of the CBL both increased. At 19∶00, these two characteristic layers were very close together. The turbulent mixing in the CBL developed by the surface heating may have intersected with the downward intrusion of stratospheric air caused by the tropopause fold (shown later).

**Figure 2:**
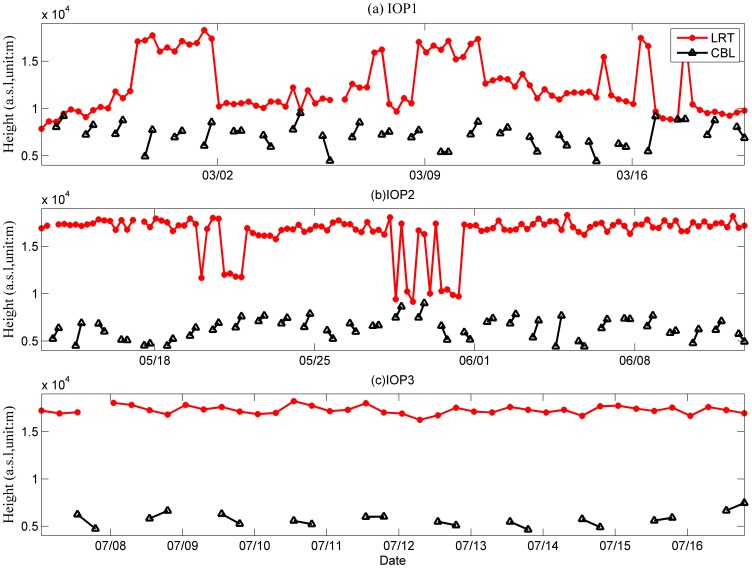
Variation of CBL depth and height of LRT during the three IOPs.

Figure 2 shows variations of the tropopause height and the CBL depth during each IOP. The results indicate that the mixing layer can be up to 5 km deep, which is greater than other previous measured values over the plateau [3], [18], [41] and deeper than predicted [42]. The tropopause and top of the CBL were very close during IOP1 especially on 25 and 26 February, and on 4, 16, 17 and 18 March. However, these two characteristic layers can still be differentiated, and the measurements place the top of the CBL below the tropopause in each case.

During IOP2 and IOP3, the potential temperature in the mixing layer was also well mixed by turbulence. The height of the CBL was lower than that in IOP1. On most days during IOP1, the height of the tropopause was below 12 km, while during IOP2 and IOP3 the tropopause height was usually around 17 km. In the afternoons, the top of the CBL ranged from 5–10 km ASL during IOP1, from 4.5–9 km ASL during IOP2, and from 5–7.5 km ASL during IOP3.

These results indicate that the tropopause was lowest and the CBL was deepest during IOP1, while during IOP2 and IOP3 the tropopause was higher and the CBL was shallower. Chen et al. [24] demonstrated that variations in the height of the tropopause were related to tropopause folds. In the next section, the displacement of tropopause folds were analyzed with synchronous CBL variations. For winter days with a low tropopause and a deep CBL, we use trajectory analysis to test the extent of interactions between the CBL and the UTLS over the Plateau.

### The Correspondence between Variations of Tropopause Folds and the CBL

**Figure 3:**
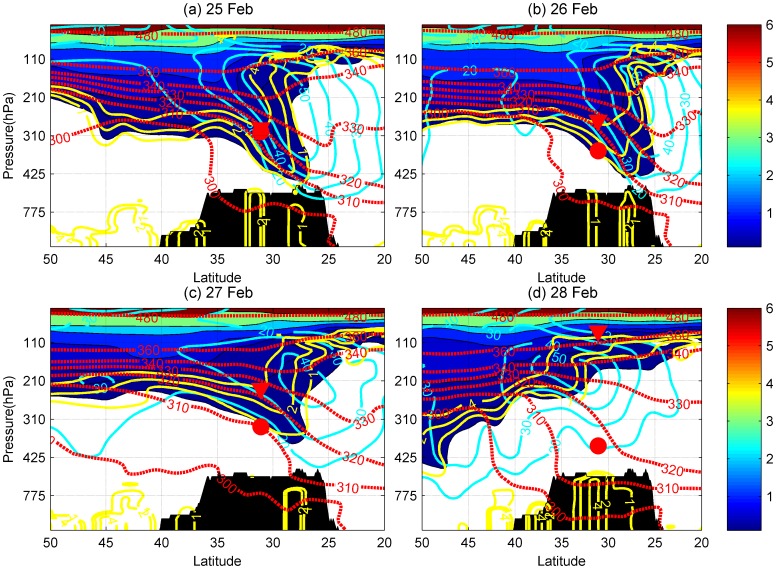
Meridional cross-section at 84.25° E (over Gerze site) at 20∶00 BST, for the period 25 Feb. to 28 Feb. 2008, derived from ERA interim data, including zonal winds (cyan contours, m/s), potential vorticity (yellow lines, contours of 1, 2, 4 PV units), ozone (solid color, ×10^6 ^kg/kg) and potential temperature (red contours, K). The color bar is the scale of ozone concentration. The area in black shows the cross section of the Tibetan Plateau terrain. The red triangles and circles show the position of the LRT and the top of CBL.

The UTLS structure around the plateau was analyzed using ERA-Interim reanalysis data, which are plotted as meridional cross sections of the atmosphere at 12∶00 GMT for 25–28 February during IOP1 (Fig. 3). The ABL is usually fully developed in the afternoon, so the ERA-Interim data at 12∶00 GMT was used here. The observed heights of lapse-rate tropopause (LRT) and top of CBL, derived from radiosonde measurements at 19∶00 BST, are labeled with red triangles and circles in the two figures. Potential vorticity (PV) isolines, zonal wind, and potential temperature derived from the reanalysis data are also shown on these graphs. The ozone content acts as a marker to indicate the tropopause fold events.

During IOP1 the westerly jet stream runs along the 28° N parallel, directly above the southern Tibetan Plateau (Fig. 3a–3d). The dynamical tropopause, identified by the isolines of 1 and 2 potential vorticity unit (PVU), exhibits a folded structure over the plateau. Several studies have revealed that wintertime tropopause folding beneath the subtropical jet stream can lead to a downward transport of stratospheric ozone into the middle troposphere, the lower troposphere or even the ABL [25], [43]–[45]. A southern downward motion on the poleward side of the jet transports stratospheric air downwards isentropically. On 25 February, the layer between 2 PVU and 4 PVU, which can be considered to represent the UTLS, reached down to 425 hPa over the Plateau and gradually decayed between 26 and 28 February. Figs. 3a and 3b indicated that the top of the CBL on 25, and 26 February was inside the tongue of the stratospheric intrusions.

**Figure 4:**
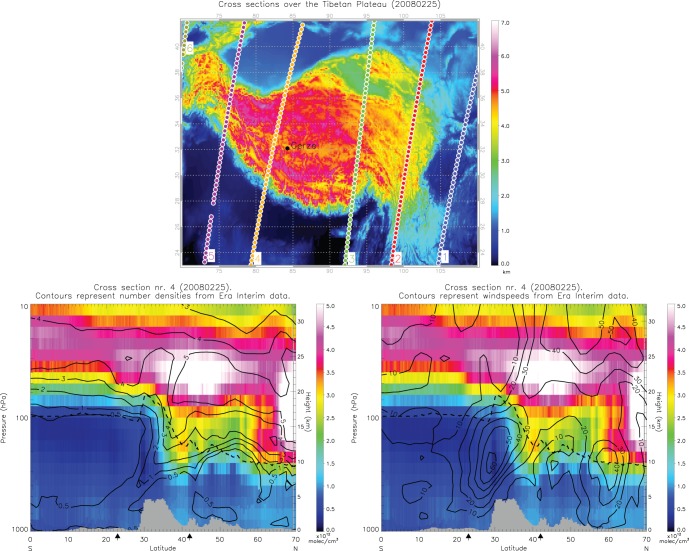
GOME-2 ozone results. Top: location of GOME-2 orbits over the Tibetan Plateau, with the altitude scale in km. Bottom left: GOME-2 ozone profiles in units of 10^12^ molecules/cm^3^, with ERA-Interim number density contours superimposed. Bottom right: GOME-2 ozone profiles with ERA-Interim horizontal wind speed contours superimposed. The arrows below the x-axis in the bottom two plots show the extent of the Tibetan Plateau as illustrated in the top plot. The dashed line is the position of the thermal tropopause, according to the WMO definition.

To validate the modeled ozone distribution derived from ERA-Interim, it was compared with data from the GOME-2 orbit closest to the Gerze station (see Fig. 4) on 25 February 2008. The observed ozone concentrations agree very well with the ERA-Interim data, and the tropopause folding event is clearly linked to the location of the jet stream over the Tibetan Plateau.

**Figure 5:**
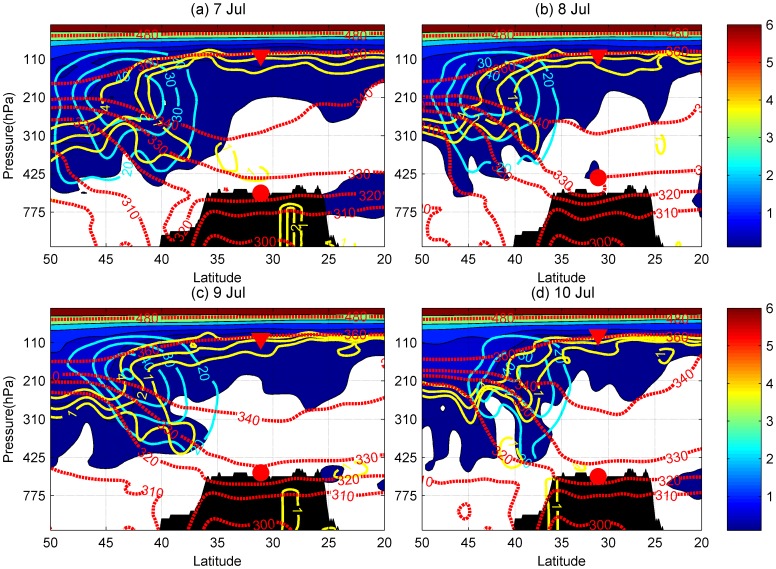
Meridional cross-section at 84.25°E (over Gerze site) at 20∶00 BST, for period 7 Jul. 2008 to 10 Jul. 2008 BST. As Figure 3, but for a period in IOP2.

During IOP3, the jet core moved north of the plateau to 42° N, as did the slightly folded UTLS structure (Fig. 5). The quasi-vertical lines of potential temperature over the Tibetan Plateau moved to the north, outside of the plateau domain. The potential temperature was more horizontally stratified over the plateau, and the troposphere was more stable during the monsoon season than during the winter time. Griffiths et al. [46] showed that the tropopause folds increase the generation of potential instability in its vicinity. When the ABL developed sufficiently to reach the height of the unstable intrusion area, it grew even higher. During the deep intrusions of 25 and 26 February the ABL was deeper than during the shallow intrusion of 28 February. During IOP3, when there were no tropopause folds, the ABL was much shallower than during IOP1.

**Figure 6:**
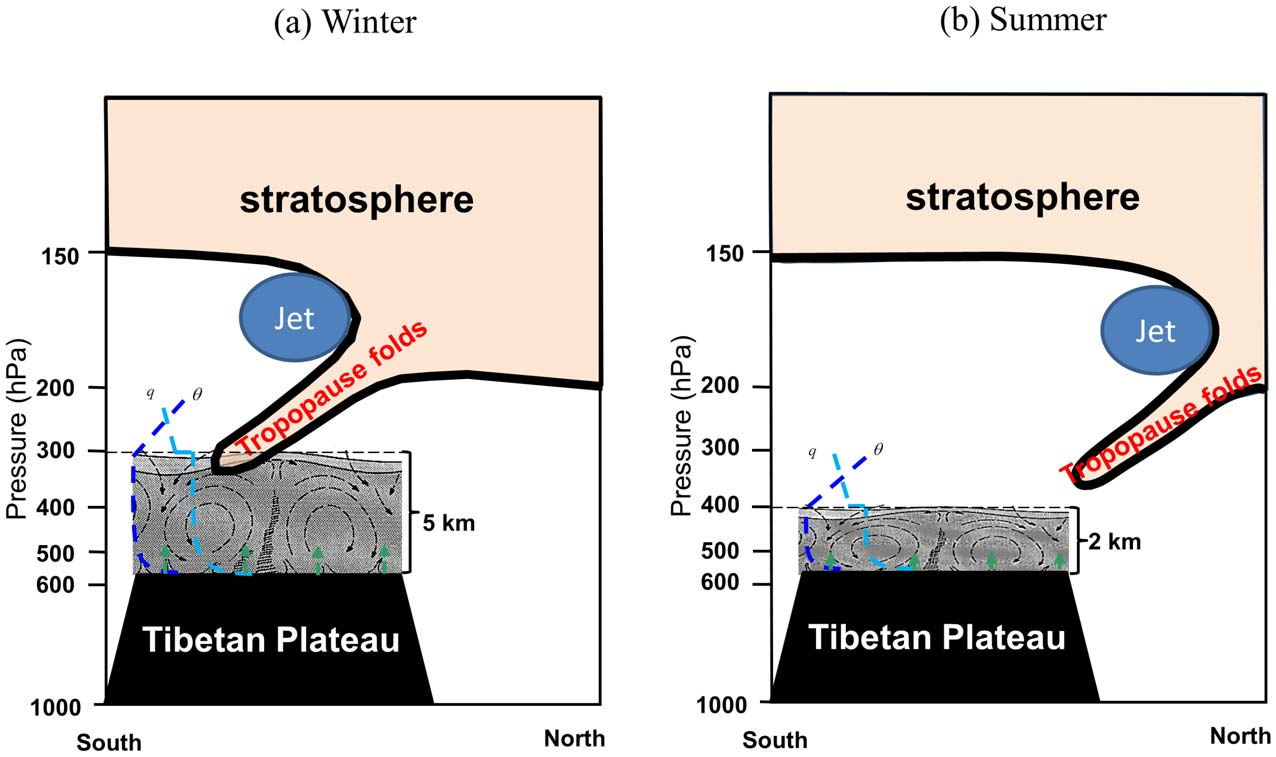
A schematic illustration of the correspondence between tropopause folds, westerly jet displacement and convective boundary layer variations during winter and summer time. The profile of water vapor (q) and potential temperature (θ) in the CBL and surface sensible heating (up green arrow) were included.

Fig. 6 gives a simple illustration of tropopause folds, westerly jet displacement and convective boundary layer variations during winter and summer time. The strongest westerly jet is situated above the plateau, with a tropopause fold below it. The frequency of stratospheric intrusions caused by tropopause folds is high during winter, which makes the upper tropospheric air unstable and facilitates ABL development. When the jet moves to the north of the Plateau and becomes weaker in summer time, the stratospheric intrusions rarely reach the upper tropospheric air above the plateau. However, whether these variations generally happen still needs more climatological study.

### Lagrangian Analysis

To gain a better insight into the relationship between a deep ABL and stratosphere and troposphere exchange (STE), trajectories of air masses for the IOP1 were computed, using the Lagrangian particle dispersion model FLEXPART v8.1 forced with ERA-Interim reanalysis data. This approach is useful for studying the origin of air masses in the UTLS [47]. The analysis was focused on IOP1, because the deep ABL and tropopause folds create a higher potential for STE.

The spatial domain used for FLEXPART was 0–180°E×0–60°N, with a vertical extent of up to 20 km, well above the top of the ABL and the tropopause. To perform the cluster analysis only the particles within a 10°×10° box around the station of Gerze at 12∶00 UTC on 25 February 2008 were used.

**Figure 7:**
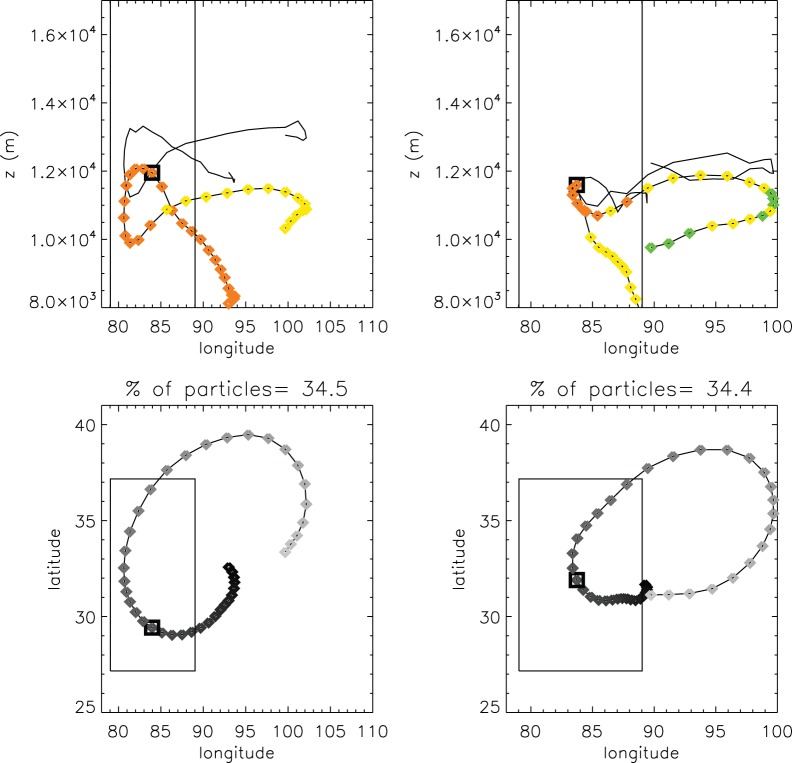
Clusters of trajectories for particles within a 10°×10° box around the station of Gerze and within the 10–14 km layer at 12∶00 UTC on 25 February 2008. The smallest square shows the central date (12∶00 on 25 Feb 2008 GMT). Each point/diamond represents one time step (3 hours). The upper plots show the height-longitude representation: the solid black line represents the height of the tropopause for each time step as calculated by the FLEXPART model, and the diamond at the lowest level corresponds to the earliest time simulated. Colors correspond to the mean PV of the air mass: orange represents 2.5–3.0 PVU, yellow 2–2.5 PVU, green 1.5–2 PVU. The lower plots show the latitude-longitude representation: the dark grey part represents earlier times in the simulation, while the light grey part represents later times.

The analysis was split by height within the reference box for the central date into three layers: 5–9 km, 9–10 km and 10–14 km ASL, which roughly represent the ABL-upper troposphere, tropopause, and lowermost stratosphere layers. The results are also clustered according to the main patterns of movement of particles determined by the model. The light grey part represents later simulated times and the dark part earlier times (see Fig. 7). The small square shows the central point (12∶00 UTC on 25 Feb 2008). Each point/diamond represents one time step (±3 hours).

The positions of most of the clusters in the three vertical layers show that the air masses stayed within the same layer for the analyzed period. We do not include the corresponding figures here. However, it is noteworthy that two clusters, accounting for a higher percentage of particles for the 10–14 km layer, show a substantial air mass from lower levels (around 8 km) and that the PV for these air masses decreased after the central date. This result indicates the possibility that air at UTLS levels is irreversibly mixed with air from the upper ABL, which can contribute to the development of a high ABL.

## Discussion and Conclusions

The structure of the troposphere over the high-altitude terrain of the Tibetan Plateau is still poorly understood, despite its impacts on regional synoptic and atmospheric circulation. Based on high-resolution radiosonde observations, we have shown measurements of a deeper boundary layer than in any previous research. The radiosonde sounding dataset of three different periods in one year demonstrates a significant seasonal contrast in ABL height. Following the suggestion of Santanello et al. [48] that atmospheric stability is the most influential variable controlling ABL development, we compared the atmospheric stability in each observation period and found that in winter time the stratification of the troposphere was related to tropopause folding events. Due to the high frequency of tropopause folds co-existing with the westerly jet situated above the plateau in wintertime [49], the isentropic surfaces in the middle and lower troposphere intersect with the Tibetan Plateau. This distribution of potential temperature lines demonstrates that the troposphere over the plateau is fairly stable during winter time, which makes it easier for a dry and warm eddy to be transported upwards. The instability associated with tropopause folds also provides a potential interpretation of the high ABL. Due to convection and vigorous vertical mixing caused by dry heating at the plateau’s surface, a dry-adiabatic lapse rate is established in the high CBL. By the afternoon, the dry thermal convection originating from the heated surface can reach the upper layers of the troposphere. A well-mixed potential temperature and water vapor layer can clearly be identified, when westerly wind dominates over the plateau. The mechanical turbulence caused by shear of the westerly wind can also contribute to the mixing of potential temperature. All these factors make the ABL much deeper, allowing the formation of larger thermals and eddies [50]. It should be pointed out that radiosonde observations are single points in time and space. We have taken radiosonde data from one station to be representative of the desired area. The mixing height is also influenced by advection, radiation and ABL cloud, which have been ignored here. Indeed, due to the low air density and intense solar radiation, the ABL of the plateau has the potential to grow much deeper than the ABL over lowlands [3]. Thermodynamic sounding profiles suggest that the direct heating of the Tibetan Plateau during winter daytime can rise to as high as 5 km above ground. These results call for a reinterpretation of the response of the ABL over the plateau to uplifted surface heating and the regional meteorological situation.

By comparison with deserts and other arid regions that also have a high ABL [51]–[53], the elevation of the Tibetan Plateau makes it easy to demonstrate how transport processes in the UTLS over the plateau affect the ABL. A close surface-troposphere-stratospheric coupled system may exist over the plateau. It has been already pointed out that the ozone flux from stratosphere to troposphere to ABL in spring is greatest over the Tibetan Plateau [54]. Johnson and Viezee [25] identified four mechanisms governing the fate of stratospheric air injected into the lower troposphere. Their mechanism 2 is a stratospheric intrusion down to the ABL, and the lower portion of the intrusion is mixed down to the ground by turbulent eddies and convection at the top of the boundary layer. Our observations show that the top of the CBL on 25 and 26 February was connected with the stratospheric intrusions, which is a typical illustration of mechanism 2. In this case, air from the lower troposphere can be mixed by turbulence and transported upwards to the tongues of the intrusions. The intrusions can also transport ozone downwards into the ABL, even down to the ground level. It has been pointed out that the strong vertical mixing in the daytime CBL can bring upper-level ozone downward to augment surface ozone production [55], [56]. Therefore, the connection between a deep CBL and the UTLS is important to the plateau’s surface ozone pollution, which is already considered as a dominant factor in diurnal variation of surface ozone in America [57], [58]. Wang et al. [59] suggested that the surface high-ozone events in the northeastern Tibetan Plateau were mostly caused by the downward transport of upper tropospheric air. The deep ABL and its coupling to the UTLS are also significant to the vertical distribution of ozone over the Tibetan Plateau.
